# Spatial Analysis of HIV Positive Injection Drug Users in San Francisco, 1987 to 2005

**DOI:** 10.3390/ijerph110403937

**Published:** 2014-04-09

**Authors:** Alexis N. Martinez, Lee R. Mobley, Jennifer Lorvick, Scott P. Novak, Andrea M. Lopez, Alex H. Kral

**Affiliations:** 1 Department of Sociology and Sexuality Studies, San Francisco State University, 1600 Holloway Avenue, San Francisco, CA 94132, USA; 2 School of Public Health and Andrew Young School of Policy Studies, Georgia State University, P.O. Box 3982, 1 Park Place, Atlanta, GA 30303, USA; E-Mail: lmobley@gsu.edu; 3 Urban Health Program, RTI International, 351 California Street, Suite 500, San Francisco, CA 94104, USA; E-Mails: jlorvick@rti.org (J.L.); snovak@rti.org (S.N.); alopez@rti.org (A.L.); akral@rti.org (A.H.K.)

**Keywords:** local indicator of spatial association (LISA), HIV/AIDS, injection drug use, San Francisco, longitudinal, GIS

## Abstract

Spatial analyses of HIV/AIDS related outcomes are growing in popularity as a tool to understand geographic changes in the epidemic and inform the effectiveness of community-based prevention and treatment programs. The Urban Health Study was a serial, cross-sectional epidemiological study of injection drug users (IDUs) in San Francisco between 1987 and 2005 (N = 29,914). HIV testing was conducted for every participant. Participant residence was geocoded to the level of the United States Census tract for every observation in dataset. Local indicator of spatial autocorrelation (LISA) tests were used to identify univariate and bivariate Census tract clusters of HIV positive IDUs in two time periods. We further compared three tract level characteristics (% poverty, % African Americans, and % unemployment) across areas of clustered and non-clustered tracts. We identified significant spatial clustering of high numbers of HIV positive IDUs in the early period (1987–1995) and late period (1996–2005). We found significant bivariate clusters of Census tracts where HIV positive IDUs and tract level poverty were above average compared to the surrounding areas. Our data suggest that poverty, rather than race, was an important neighborhood characteristic associated with the spatial distribution of HIV in SF and its spatial diffusion over time.

## 1. Introduction

Two decades of intensive community-based, local HIV prevention efforts have shaped the trajectory of the HIV/AIDS epidemic among injection drug users (IDUs) in San Francisco. [1,2,3,4,5,6]. Prior to the widespread availability of sterile syringes in the late 1980s, HIV incidence among IDUs in San Francisco peaked in 1987 at a rate of 2.7% [7]. The subsequent decline in incident infections has been largely attributed to the successes of community based programs in expanding the availability of sterile syringes, condoms, and HIV testing to a marginalized population often characterized as hidden and unwilling to utilize services offered by conventional public health programs [8,9,10]. The ongoing rate of HIV transmission among IDUs in San Francisco has remained moderate and stable since 1989 [11], and efforts to reduce the rate of new infections have evolved to focus on structural factors that alter the environment in which individual behavioral risks take place [12,13]. In particular, a growing body of international research is focused on linking behavior with place and utilizing spatial analytic tools to examine salient issues relevant to HIV prevention [14,15], which include placement of HIV service providers in large urban cities, improper disposal of previously used syringes [16,17,18], and identification of geographic HIV-related risk hotspots [19,20,21,22,23]. These types of spatial analyses, which involve studying the distribution of HIV-related outcomes at the geographic level rather than the individual level, have the potential to offer new insights for HIV-related public health policy and prevention programs. 

For example, in Toronto, researchers used exploratory spatial data analysis to visualize the distribution of HIV service providers and assess the characteristics of the neighborhoods in which services are geographically concentrated [24]. The analysis showed that individuals living in neighborhoods with high levels of poverty, immigration, and residential instability had reduced access to HIV-related services. In Montreal, spatial analytic techniques were used to show that improperly discarded syringes clustered near bus stops, pay phones, pawnshops and single-room occupancy hotels (SROs) [25]. Finally, spatial analytic tools have been used to show that clusters of HIV infected individuals in Russia co-cluster with heroin injection, receptive syringe sharing, being younger than 24 years, and living with parents. [19] 

Additional spatial analysis of HIV epidemiological data is needed in large, urban metropolitan cities in the United States that have been heavily afflicted by the HIV/AIDS epidemic [14]. Previous research has shown that the racial and economic composition of neighborhoods is associated with geographic clustering of HIV-related outcomes. [19,26,27,28,29,30] In Atlanta, Georgia, researchers identified a single geographic cluster that contained 60% of prevalent HIV cases in the metropolitan area. This cluster of HIV cases was associated with higher levels of poverty and lower levels of racial/ethnic diversity. [26] In San Francisco, the first AIDS cases in San Francisco were reported in May 1981 among gay men residing in a relatively affluent neighborhood known as the Castro [31]. Geographic diffusion of the virus occurred by 1984 with the emergence of a second epicenter of AIDS cases among IDUs in a comparatively lower income neighborhood known as the Tenderloin. Ongoing transmission of the HIV virus continues to vary by neighborhood, with recent research suggesting that community viral load, or the amount of circulating virus in HIV positive individuals, is higher in neighborhoods characterized by poverty, large numbers of IDUs, high proportions of African American residents, and commercial sex workers [32].

### Purpose of Paper

This paper aims to contribute to the literature on spatial epidemiology of HIV by analyzing the geographic distribution of HIV positive IDUs in San Francisco over a 20 year time period. In this paper, we examine data aggregated to the level of the Census tract and employ Moran’s I and Local Moran’s I (LISA) tests to examine patterns in HIV seropositivity among injection drug users in San Francisco. We use a new method that has not been described in the HIV prevention literature to date, the bivariate LISA test statistic [33]. 

We use the bivariate LISA to identify geographically coincident clusters of a pair of variables simultaneously, and provide a statistical test to determine whether the coincidence is so strong that it would not likely have occurred by chance. As a second exploratory analysis, we conduct statistical tests of differences in areas included in HIV clusters *versus* areas not included in these clusters. We compare three tract level characteristics of interest across clustered and non-clustered Census tracts. The results from the two analytical approaches are compared to assess consistency of findings. 

Overall, the purpose of this paper is to illustrate changes in the geography of the HIV/AIDS epidemic in San Francisco over two decades and identify tract level characteristics that are associated with spatial clusters of HIV cases over time. 

## 2. Methods

### 2.1. Urban Health Study (UHS)

The UHS was a serial, cross-sectional epidemiological study. For every observation in the dataset starting in 1987, there exists geocoded data denoting where the participants “usually stay”. Participants were asked where they “stay” rather than where they “live” because of the high prevalence of homelessness in the sample [34]. Data were collected every 6 months using targeted sampling methods. Targeted sampling is a purposeful and systematic method for sampling hidden and uncountable populations. Between 1,400 and 2,600 observations were incorporated into the data set each year. The combination of the study’s serial cross-sectional design and the use of targeted sampling methods ensured that the sample was as representative of the IDU community in San Francisco at each cross-sectional period as possible, given that randomization was not feasible [35]. A key strength of targeted sampling is its responsiveness to changes in the IDU community over time, as described below. Our data collection was highly responsive to changes in locations of IDUs and helped ensure that our field sites were in the most convenient locations for the majority of IDUs in the study communities.

The procedures for sampling, recruitment, intake, eligibility screening, informed consent, quantitative interviewing, and HIV testing were kept uniform throughout the study period. Data were collected in neighborhoods at easily accessible community field sites, such as churches, single room occupancy hotels, and community centers. The locations of field sites were selected each year to be near the highest density of IDUs. Eligibility criteria for initial entry to the study were uniform throughout the study: (1) injection drug use in past 30 days, or previous study participation; (2) ability to provide informed consent; and (3) age 18 or older. Upon presentation to the study site, first-time participants were screened for physical evidence of chronic venipuncture consistent with multiple drug injections (“tracks”). Previous participants were allowed to participate once every 6 months, regardless of whether they continued injecting. 

A structured quantitative survey was administered to each participant by trained interviewers. Items were read aloud and responses recorded by the interviewer. Variables consistently collected over the life of the study include gender, race, age, sexual orientation, use of each type of injection and non-injection drugs (e.g., heroin, cocaine, methamphetamine), alcohol use, syringe sharing, sexual partners (including number and gender), use of drugs by steady sexual partner, sex work, condom use by type of sex (vaginal, anal, oral), drug treatment utilization, arrests and incarceration history, and HIV testing history. Following completion of the interview, 40 mL blood samples were obtained by phlebotomy staff from each participant. Blood specimens were analyzed using enzyme immunoassay (EIA) according to CDC guidelines. Repeatedly EIA-positive specimens were confirmed using Western blot assay. We analyzed HIV test result data using a dichotomous variable that equaled 1 if participants tested positive at the time of interview and equaled 0 otherwise. After deleting all observations with missing geographic or HIV test results, the final number of participants in the analytic sample was 23,276 interviews representing 174 Census tracts.

### 2.2. Random Data Accuracy Check

Two hundred paper UHS surveys were chosen at random to assess the accuracy of the geocoding performed by the data entry organization used throughout the study period. Each paper survey included the original intersections reported by the participant as usual place of residence. A research assistant entered the intersections into the United States Census website to double check the accuracy of the geocoded tract. Of the 200 surveys chosen at random, 92% was accurately geocoded to the correct census tract. 

### 2.3. Census Tract Measures

We abstracted data from the U.S. Census Neighborhood Change Database to develop the Census tract level measures using “normalized” U.S. Census tract data from 1990 and 2000. We merged the census tract characteristics from the Neighborhood Change Database with the individual level UHS data by census tract. Based on our review of the literature on neighborhood effects and HIV/AIDS among IDUs, we included three census tract level variables in our analysis [25,36,37,38]. The three census tract variables were: (1) proportion of persons living under the poverty level, (2) proportion of unemployed persons still actively seeking employment, and (3) proportion of African Americans. All of these variables have been previously shown to be associated with HIV risk or prevalence at the neighborhood level. [30,39,40,41] The poverty level for United States 2000 is defined as a set of money income thresholds that vary by family size and composition to determine who is poor [42]. For example, a family of four, with two related children under the age of 18, will count as poor if the total family income is less than $17,463. For the unemployment variable, unemployed civilians are those over 16 who did not have a job during the reference period, were actively looking for work, or waiting to be called back to a job from which they had been laid off, and were available to go to work [43]. According to the U.S. Census, the Black or African American refers to a person having origins in any of the Black racial groups of Africa. It includes people who indicated their race(s) as “Black, African Am., or Negro” or reported entries such as African American, Kenyan, Nigerian, or Haitian [44].

### 2.4. Early and Late Time Period

The two time periods in this analysis reflect an “early period” (1987 to 1995) and a late period (1996–2005). The HIV/AIDS epidemic underwent a profound shift in 1996, the year that antiretroviral medications became available in the United States. [45] The era of antiretroviral medications in the U.S. allowed HIV positive persons to reduce their viral load and slow the development of opportunistic infections that characterize AIDS. Mortality rates declined steeply and rates of HIV testing increased following the availability of antiretroviral medications. We use this year to cut our data into two periods, reflecting before and after a change in availability of medication, representing a natural experiment that allows us to assess changes in the spatial distribution of HIV positive injection drug users over this 20 year period in San Francisco.

### 2.5. Tests of Spatial Association

For each time period we analyzed total counts of HIV positive IDUs in Census tracts of residence. We performed both global and local, and univariate and bivariate tests of spatial autocorrelation to determine the presence of spatial clustering [46]. For the univariate tests, we conduct a global test (Moran’s I) followed by a set of local tests (LISA) [47]. The global test hypothesizes that there is a spatially random pattern of HIV positive IDUs among census tracts, which is rejected in the presence of any local spatial clustering. To see exactly where this local clustering occurs, the local indicators of spatial association (LISA) tests are used [48]. Four types of spatial autocorrelation are observed using the LISA statistic (high-high, low-low, high-low, and low-high). Positive spatial autocorrelation (*i.e.*, an association of areas of similar values) is represented by high-high and low-low, and negative spatial correlation (*i.e.*, an association of areas of dissimilar values) by high-low and low-high. A finding of significant clustering at *p* < 0.05 suggests that values for observed counts of HIV positive IDUs are too similar across these neighboring Census tracts to have occurred by chance, providing significant evidence for rejecting the null hypothesis of spatial randomness. Because population estimates of IDUs by census tract do not exist [49], we opted not to calculate an HIV rate in each census tract. Instead we used counts of HIV positive IDUs in each tract to perform the LISA tests.

The bivariate LISA gives an indication of the degree of linear association (positive or negative) between the values for two variables: one variable at a given location and the average of another variable at its neighboring locations. We use the bivariate LISA to assess correlation between counts of HIV positive IDUs and three different contextual variables of interest, for each tract and its neighboring tracts. Specifically, we look at the correlation between X (HIV) at each location and average Z (e.g., poverty) in the surrounding locations. 

The association among locations for either the local (LISA) or global (Moran’s I) tests is defined using a spatial weights matrix. The weights matrix describes for each location all of the closest neighboring locations and is derived either from a distance matrix that contains distances between all possible pairs of locations, or a contiguity matrix that assesses common boundaries. We used a queen contiguity weights matrix that defines a location’s neighbors as those with either a shared border or vertex/corner. We identify significant clusters by mapping the individual tracts that make up the cluster. The cluster maps in Figure 2, Figure 3, Figure 4 and Figure 5 (see Section 3.3 and Section 3.4) show the *center* of the cluster in color (e.g., red for a high-high cluster). The actual extent of the cluster includes the center and the surrounding neighbors as defined by the weights matrix.

Spatial analyses were conducted using GeoDa software [48] and results were mapped using ArcView GIS (geographic information systems) software (ESRI, Redlands, CA, USA). We calculated a Global Moran’s I statistic for counts of HIV positive IDUs in both time periods, 1987–1995 and 1996–2006. We found global evidence of clustering and then conducted univariate LISA tests in each period to see exactly where the spatial clusters were located. We then conducted bivariate LISA tests in each period to determine if any of the three contextual variables of interest were spatially associated with HIV clusters. The contextual variables of interest at the census tract level included percentage of households living under the poverty level, percentage unemployment, and percentage African Americans. The LISA statistic is calculated for each observation and clusters that are significant at the *p* < 0.05 level, including the tracts that make up the center of the cluster and the contiguous neighbors, are highlighted in Figure 2 and Figure 3 (univariate) and Figure 4 and Figure 5 (bivariate). 

We expect that findings from the bivariate LISA approach will be consistent with a more ad-hoc *t*-test approach conducted by comparing means of key variables in the clustered and non-clustered tracts identified using the univariate LISA. If these contextual variables contributed to the observed spatial clustering in counts of HIV positive IDUs, we would also expect to find significant differences in mean values of these contextual variables across tracts composing clusters with high counts of HIV positive IDUs and other non-clustered tracts. Following Mobley *et al*. [50], we assessed the significance of differences between the means of these two groups of contextual variables using a paired *t-*test for each variable (IBM SPSS Version 20, Armonk, New York, USA).

## 3. Results

### 3.1. Descriptive Statistics of IDUs by Census Tract for the Early and Late Period

The number of study participants grew over time, from 11,538 in the early period to 16,074 in the late period. For the early time period, there were no study participants in 50/174 (29%) Census tracts. The distribution of participants by census tract is highly skewed, ranging from a minimum of 1 participant per tract to a maximum of 1,360 participants per tract. The mean number of participants per tract is 66 with a standard deviation of 180, and the median is 4 participants per tract with an interquartile range of 0 and 39. For the late time period, there were no study participants in a smaller proportion of Census tracts, 22/174 (12.6%). Like the early period, the distribution of participants is highly skewed, ranging from a minimum of 1 participant per tract to a maximum of 1,136 participants per tract. The mean number of participants is 76 per tract with a standard deviation of 184, and the median is 9 with an interquartile range of 2 and 45. These descriptive statistics show that over time, IDUs spread out across more tracts in San Francisco.

### 3.2. Socio-demographic Profiles of IDUs in Census Tracts for the Early and Late Period

**Figure 1:**
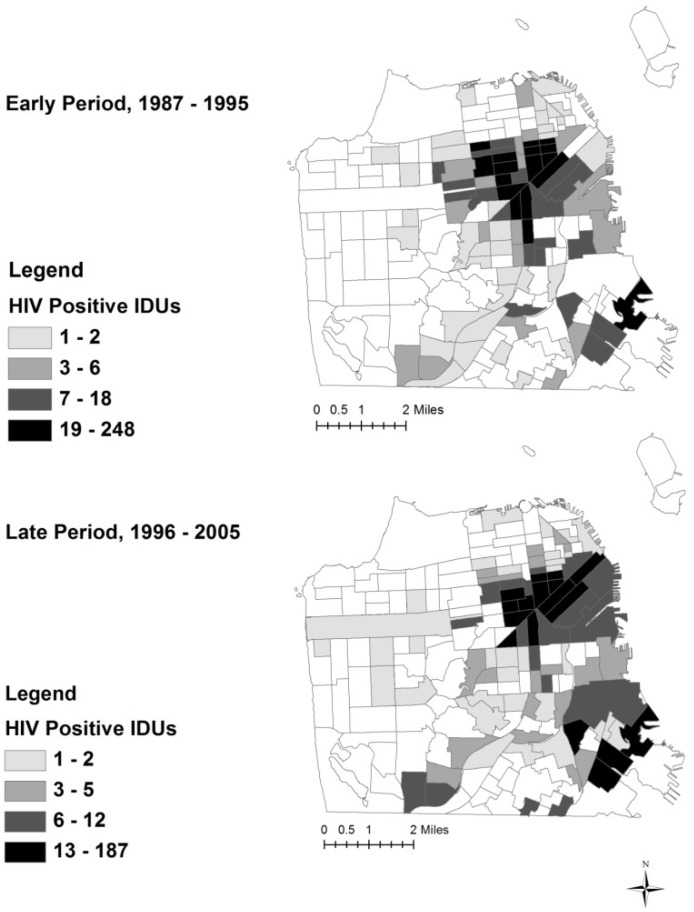
Quartile Map of HIV Positive Injection Drug Users in San Francisco.

Table 1 and Table 2 describe UHS data in 2 different ways. Table 1 presents characteristics of study participants for the entire time period (1987–2005). Data in Table 1 showed that the highest proportion of observations consisted of male, White, heroin injectors with a lengthy history of injection and high prevalence of service utilization, including syringe exchange and drug treatment. 12.8% of all observations tested HIV positive at the time of interview. Table 2 presents Census tract averages of IDU characteristics. Mean HIV prevalence in a Census tract, defined as the average of HIV positive IDUs divided by the total number of IDUs in each Census tract, is 14% in the early period and 10.2% in the late period. Of the 174 Census tracts in San Francisco, 49% (n = 86) of tracts had at least one HIV positive IDU in the early period, and 50.5% (n = 88) of tracts had at least one HIV positive IDU in the late period. Quartile maps of HIV positive IDUs in the early and late period are shown in Figure 1. 

The mean percentage of white IDUs in a Census tract increased between the early period and the late period whereas the mean percentage of African Americans and Latinos decreased. 

**Table 1:** Characteristics of UHS Sample from 1987 to 2005.

Variable	N = 29,914
*Gender*	%
Male	67.9
*Age*	
Under 30	7.6
30–49	71.7
50 or older	17.5
*Race/Ethnicity*	
White	55.3
African American	29.3
Latino	7.3
Other	0.9
*Socioeconomic status*	
Homeless	36.9
HIV-positive	12.8
*Drug use (30 days)*	
Injected heroin	73.1
Injected methamphetamine	22.7
Smoked crack cocaine	53.8
Mean # years injecting	22 years
*Drug treatment*	
Ever in drug treatment	76.0
*Service utilization*	
Syringe exchange past 6 months	69.8
*Injection risk past 6 months*	
Any receptive or distributive syringe sharing *	18.7

Note: * Data only available from 1992–2005.

**Table 2:** Tract Averages for the Early and Late Period.

Variable	1987 to 1995 Mean %	1996 to 2005 Mean %
*Gender*		
Male	67.0	69.5
*Age*		
Under 30	14.8	11.0
*Race/Ethnicity*		
White	31.6	46.1
African American	46.7	35.3
Latino	12.7	7.3
*Socioeconomic status*		
Homeless	29.3	43.9
Arrested in past year	53.5	27.8
*Health status*		
HIV-positive	13.7	10.2
*Drug use (6 months)*		
Injected heroin	70.2	74.0
Injected methamphetamine	15.4	24.4
Smoked crack cocaine	42.7	53.9

The mean percentage of African Americans in a tract went down from 46% to 35%, suggesting a geographic dispersion of African Americans across the census tracts that had IDUs in them. The percentage of homelessness among IDUs at the tract level dramatically increased from the early to late period. Specific to drug use behaviors, we observed no change in the mean percentage of IDUs who reported heroin use, and a small increase in the mean percentage of methamphetamine use for all census tracts in which IDUs lived between 1996 and 2005. 

### 3.3. Univariate Tests of Spatial Association

**Figure 2:**
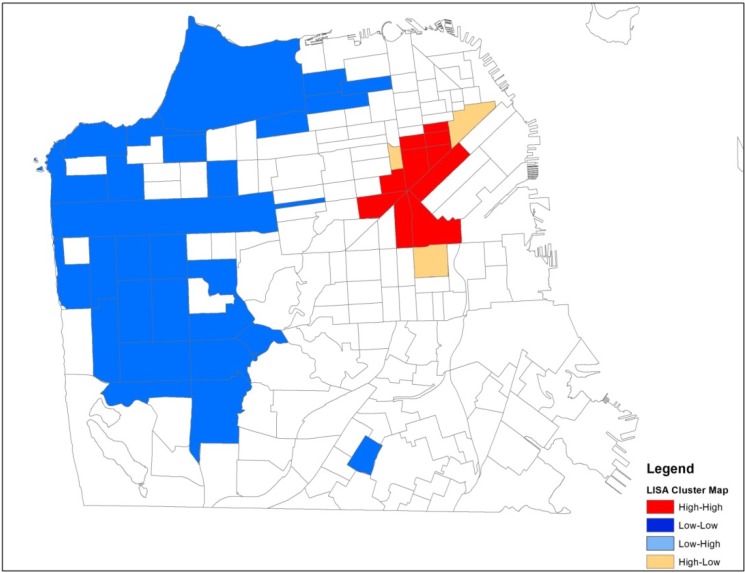
Spatial patterns of HIV positive injection drug users, 1987 to 1995.

**Figure 3:**
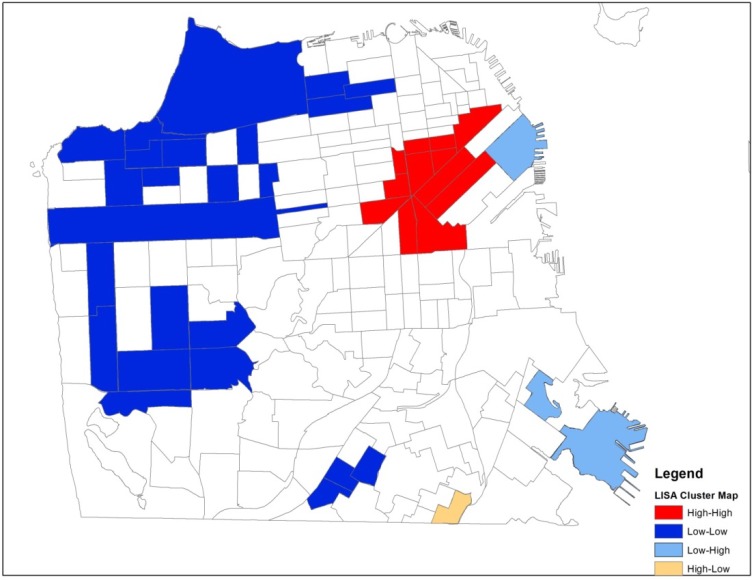
Spatial patterns of HIV positive injection drug users in San Francisco, 1996 to 2005.

The Global Moran’s I statistic is statistically significant in both periods (0.28 in the early period and 0.38 in the later period), suggesting local clustering somewhere in San Francisco in both periods. The Global Moran’s I statistic is greater in the late period reflecting the geographic dispersion of this population over time. To identify areas of local clustering, we used GeoDa software to generate a LISA cluster map that shows four types of geographical clustering (high-high, low-low, high-low, low-high). We focus on areas of significant high-high clustering that show Census tracts with above average counts of HIV positive IDUs that share boundaries with neighboring Census tracts that also have above average values of HIV positive IDUs. In the early period and the late period, we observed high-high clustering of HIV positive IDUs in downtown commercial neighborhoods of San Francisco (Figure 2 and Figure 3). 

The area of high-high clustering in the early period spanned 30 Census tracts, including 10 tracts that make up the center of the cluster and 20 neighboring tracts. The geographic area of high-high clustering in the late period expanded to 36 Census tracts, including 12 tracts that make up the center of the cluster and 24 neighboring tracts. The area of high-high clustering in the late period accounted for 12.5% of all HIV positive IDUs in the sample. In the early period, the mean tract HIV prevalence in the significant high-high clustering area was 16.2%. The mean HIV prevalence outside the area of high-high clustering was 9.8%.In the late period, the mean HIV prevalence of the tracts that were part of the significant high-high clustering area was 14.7%. The mean HIV prevalence outside the area of high-high clustering was 8.5%.

### 3.4. Bivariate LISA

**Figure 4:**
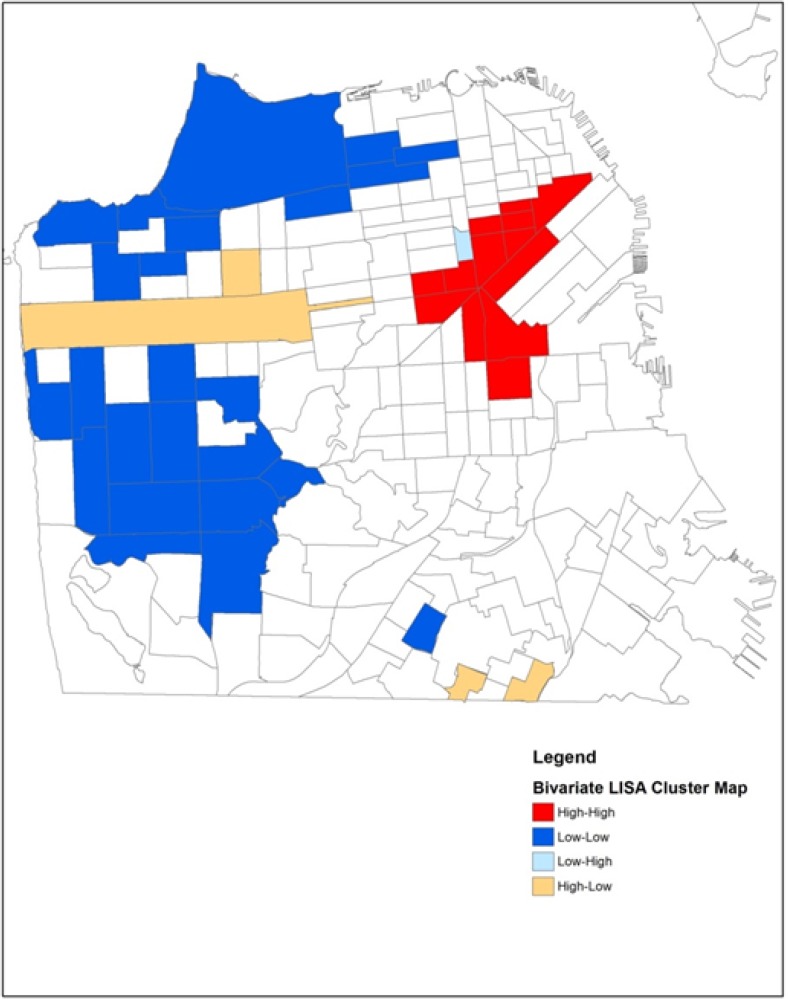
Significant bivariate clusters of high HIV counts and high poverty rates, 1987 to 1995.

**Figure 5:**
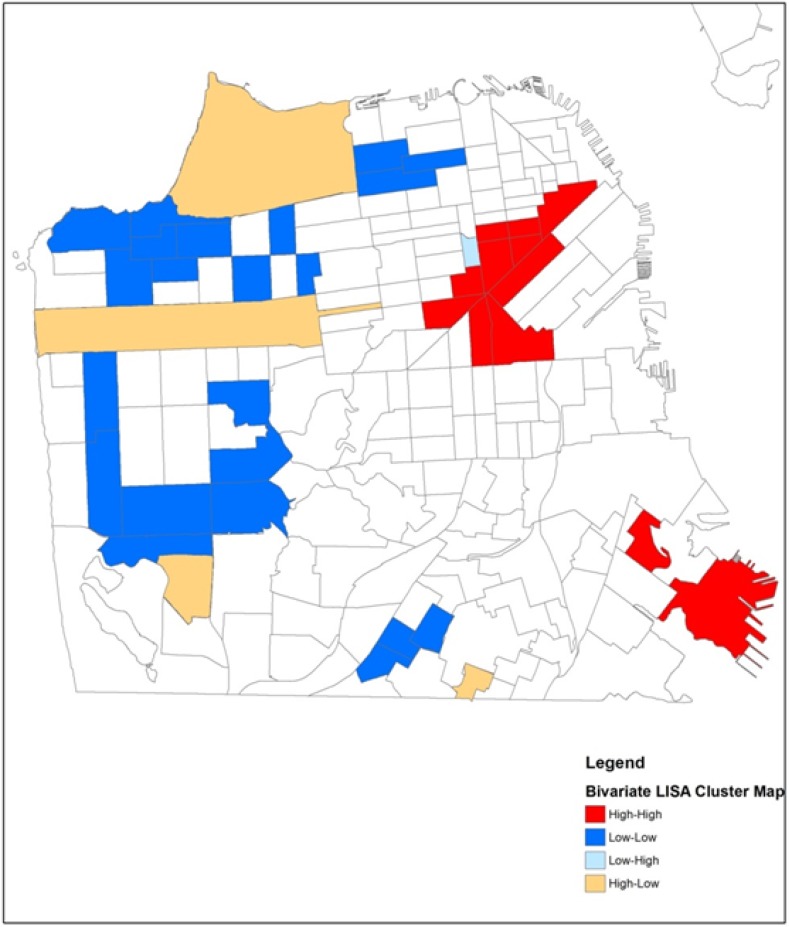
Spatial high HIV counts and poverty rate using tests of local indicators of spatial autocorrelation, 1996 to 2005.

The bivariate test provides information regarding contributors to the observed geospatial patterns of HIV positive IDUs and where these associations are strongest. This bivariate test identifies Census tracts where counts of HIV positive IDUs and poverty are above average compared to the surrounding areas. In both time periods, significant bivariate clusters were found for both poverty and unemployment (paired with HIV) but not for percent African American in tracts. Comparing the bivariate results for poverty presented in Figure 4 and Figure 5 with the univariate results presented in Figure 2 and Figure 3, we see that the bivariate LISA suggests a different pattern for concentration of HIV positive study participants in San Francisco’s neighborhoods over time, when simultaneously considering neighborhood poverty. In the late period there are two distinct positive-positive clusters where counts of HIV positive IDUs and poverty rates in Census tracts are above average compared to the surrounding areas. 

### 3.5. Robustness Check

Following the approach used in Mobley *et al* [50], we used a simple t-test to assess whether there were significant differences in average values for the three contextual variables of interest across clustered and non-clustered locations identified in the univariate LISA analysis (Table 3). There was a significantly higher percentage of households in poverty in high-HIV clustered areas compared to all other locations in both the early period (21% *vs*. 12%) and the late period (17% *vs*. 10%). A significantly higher mean unemployment rate was also found in the high-HIV clusters than in other places, in both periods. However, no significant difference was found in the percent African American in either period. This pattern of significance in the contextual factor means across clustered and non-clustered areas is consistent with the findings from the bivariate LISA tests. 

The bivariate *t*-test suggests that HIV clustered and non-clustered tracts are globally differentiated by poverty. The bivariate LISA shows exactly where the correlation between HIV clustering and poverty is the strongest. The bivariate LISA is more refined than the ad-hoc t-test approach, because it allows statistical assessment of the significance of the correlation between context and HIV cases at each location, whereas the *t*-test approach is a global test of this correlation across all clustered and non-clustered places. The *t*-test also assumes that these two groups of places are statistically independent samples, which we know (from the global and local tests of spatial association) to be a false assumption. 

**Table 3:** Means (SD) and Medians (IQR) of Census Tract Variables among high HIV clusters and neighbors and all other locations.

Census Tract Variables	Mean (SD)	Mean (SD)	Median (IQR)	Median (IQR)
**Early Period** **(1987–1995)**	**n = 23**	**n = 151**	**n = 23**	**n = 151**
% of households living under the poverty level	0.21 (0.08) *	0.12 (0.11)	0.21 (0.17, 0.28)	0.09 (0.06, 0.15)
% of unemployed persons still in labor force	0.13 (0.12) *	0.06 (0.04)	0.10 (0.06, 0.15)	0.05 (0.04, 0.08)
% of African Americans	0.16 (0.18)	0.11 (0.17)	0.08 (0.05, 0.24)	0.04 (0.06, 0.11)
**Late Period** **(1996–2005)**	**n = 32**	**n = 142**	**n = 32**	**n = 142**
% of households living under the poverty level	0.17 (0.07) *	0.10 (0.07)	0.17 (0.10, 0.21)	0.08 (0.06, 0.11)
% of unemployed persons still in labor force	0.07 (0.05) *	0.05 (0.04)	0.06 (0.04, 0.08)	0.04 (0.03, 0.05)
% of African Americans	0.10 (0.11)	0.08 (0.14)	0.06 (0.03, 0.15)	0.03 (0.01, 0.08)

Note: * *p*-value < 0.05.

## 4. Discussion

The analyses presented in this paper make a substantive methodological contribution to the growing body of spatial research on the HIV/AIDS epidemic. Our visualization of the residential locations of HIV positive injection drug users (IDUs) in San Francisco over a 20 year period showed that the study sample became more geographically dispersed over time. In the early period, there were 3 tracts with higher than average HIV positive IDUs that were bordered by tracts with lower than average HIV positive IDUs (high-low clusters, Figure 2). In the late period, two of these 3 tracts had become part of a significant cluster of above average HIV positive IDUs. Using the bivariate LISA analysis, we found that new areas of HIV clustering appeared in the late period in the Bayview-Hunters Point, a historically African American and geographically isolated area in the southeastern part of San Francisco. While the study sample of IDUs became more geographically dispersed over time, they remained concentrated in tracts with high levels of poverty. Although African American and poverty variables are generally known to be highly correlated in the social sciences literature, in San Francisco at the time of the Census 2000, the tract level percent African American and percent households in poverty had a simple correlation of only 0.341. Thus there were many tracts with high percent poverty but low percent African American residents. Our data suggests that socioeconomic status, rather than being African American, was an important neighborhood characteristic associated with the spatial distribution of HIV in San Francisco during both study periods, and its spatial diffusion over time. 

Methodologically, we apply and explain the importance of the bivariate LISA test [51], which has not been previously used in the field of HIV/AIDS research. This paper provides a context for understanding this somewhat complex statistical test of spatial association. We argue that the bivariate LISA is a more sophisticated way to assess patterns in contributing variables across clustered and non-clustered places than some more *ad-hoc* approaches seen in the literature to date. [26,50] These *ad-hoc* approaches are global in nature and do not account for spatial autocorrelation, which may bias the estimated standard errors and levels of statistical significance. On the other hand, the LISA tests have been criticized for failure to account for multiple testing in the reported p-values. That is, each local test is conducted independently of the tests for each other locations, while all draw from the same data in creating the statistical distribution for the test statistics. Thus neither approach is perfect. 

In our data, we find consistency between the two approaches in terms of which geographic characteristics are important contributors to clusters in outcomes. The main advantage of the bivariate LISA approach is the ability to assess statistical significance of the bivariate relationships at specific locations. Thus, in the same way that the local Moran’s I (LISA) provides more specific information than the global Moran’s I, the bivariate LISA provides more specific information than *ad-hoc* approaches that globally compare characteristics of clustered and non-clustered places. Bivariate LISA tests also allow for statistical assessment of significance of bivariate patterns, an advantage over more qualitative comparisons of univariate clusters identified independently for related variables. Bivariate LISA tests are limited to bivariate comparisons, while regression approaches can assess multiple covariates across clustered and non-clustered places.

## 5. Limitations

Since injection drug use is a clandestine and illegal activity, it is not possible to conduct random sampling of the study population. This means that there may be limited generalizability of results obtained from studies of IDUs. The purpose of using a targeted sampling approach to recruit IDUs in the UHS was to generate a more representative sample of IDUs than might be obtained from dependence on institutional samples. We found the spatial distribution of IDUs to be very similar among UHS participants, entrants to drug treatment, and people arrested for drug-related crimes, suggesting that our sample is as representative as can be achieved given these limitations. Nevertheless, potential sources of bias exist. The UHS relies on self-report of socially undesirable behaviors, which may result in response bias, although UHS was an anonymous study, which likely reduced concern about reporting socially undesirable behaviors. Studies of IDUs in non-institutional settings have shown to have high reliability and validity of self-reported information [52,53]. Another potential source of bias derives from misclassification of census tract information. We relied on self-report of intersections from individuals who, with a history of illicit drug use, may have been unwilling to disclose this information. In addition, many of our study participants had unstable housing situations. We do not know whether each study participant usually stayed in the same census tract over the full 6-month period. While it would be ideal to conduct a longitudinal study that followed individual injection drug users who were exposed to different neighborhood conditions, such a study would be prohibitively expensive given the need for large sample sizes in this type of research and excellent retention rates. This dataset allows us a unique opportunity to begin assessing spatial patterns among HIV positive IDUs in San Francisco over the past two decades. 

## 6. Conclusions

The policy and prevention implications of a spatial approach to studying HIV/AIDS are numerous. First, placing HIV prevention services, such as syringe exchange programs and drug treatment programs, where they are needed most is critical to efficiently allocate scarce public health resources. Identifying spatial clusters of HIV positive IDUs can inform that process. Future research that utilizes spatial data should also focus on HIV risk behaviors, such as syringe sharing, for the purpose of identifying clusters of risk rather than prevalence. Second, identifying historical IDU population shifts between neighborhoods that also underwent changes in poverty is important to consider in the context of future changes to the economy of San Francisco. Public health departments might anticipate shifts in HIV prevalence from one geographic area to another and be able to respond with the spatially targeted tools and resources. Overall, observing spatial relationships between HIV-related outcomes and characteristics of a neighborhood may also lead to important questions about the neighborhood itself as a site of future research, in terms of changing identities, boundaries, and resources that may be relevant to HIV prevention efforts. Finally, it is important to note that moving forward with this type of spatial research requires the collection of geographic data that links an individual’s behavior to a physical location, and therefore necessitates considerations of confidentiality and privacy for those who already find themselves in structurally vulnerable positions within society.
